# Soft tissue expansion using self-inflating osmotic hydrogel expanders prior to bone augmentation: healing and complications. Evidence-based review

**DOI:** 10.1038/s41405-023-00175-3

**Published:** 2023-11-11

**Authors:** Adam Gade Ellesøe, Rawand Shado, Ines Novo Pereira, David Madruga, Haidar Hassan

**Affiliations:** 1 Rey Juan Carlos University, Av. de Atenas, S/N, 28922 Alcorcón, Madrid Spain; 2 Barts & The London School of Medicine & Dentistry, Queen Mary University, Institute of Dentistry, Royal London Dental Hospital, Turner Street, E1 2AD London, UK; 3 University of Porto, Faculty of Dental Medicine, R. Dr. Manuel Pereira da Silva, 4200-393 Porto, Portugal; 4 Barts & The London School of Medicine & Dentistry, Queen Mary University, Centre for Cutaneous Research, Blizard Institute of Cell and Molecular Science, 4 Newark Street, Whitechapel, London, E1 2AT UK

**Keywords:** Dental implants, Resorption, Dentoalveolar surgery, Dental implants

## Abstract

**Aim:**

This review aims to assess complication rates, soft tissue gain, and bone gain associated with the use of self-inflating osmotic hydrogel tissue expanders (SOHTEs) for soft tissue expansion (STE).

**Methods:**

A comprehensive search on Pubmed and Google Scholar databases was conducted to identify human studies using SOHTEs for STE; last searched in March 2023. Expansion phase details and expander variables were documented. Complication rates, soft tissue gain, and bone gain reported in each study were also recorded. The inclusion criteria encompassed human studies ranging from evidence levels II–IV (Oxford Centre for Evidence-Based Medicine Levels of Evidence), without specific date limits. For assessing bias in randomized controlled trials (RCTs), a Risk of Bias tool was employed. The synthesised results were presented through tables, sunburst plots, and bar charts.

**Results:**

A total of 13 studies were identified, comprising 4 RCTs, 1 cohort study, and 8 case-series. Employment of SOHTEs yielded an overall complication rate of 17% (24/140 sites), with expander perforation accounting for 9.3% (13/140) of the sites. Specific complication rates included dehiscence (1.4%, 2/140 sites), paraesthesia (1.4%, 2/140 sites), and infection (1.4%, 2/140 sites). All randomized controlled trials (RCTs) were categorised at Level II. The remaining investigations primarily consisted of Level IV case-series lacking controls. All studies demonstrated some concerns towards bias.

**Conclusion:**

STE studies using SOHTEs exhibit a reduction in complications associated with bone augmentation in scenarios of inadequate soft tissue coverage. Preliminary evidence suggests potential benefits even in cases with sufficient soft tissue. Adherence to procedural precautions may reduce the risk of expander perforations, further diminishing complications. Subsequent studies should incorporate individual patient and expander variables in their reports to explore the impact of expansion phases on complication rates, as well as bone and soft tissue augmentation.

## Introduction

### Reasons for bone augmentation

The absence of an adequate bone necessitates a bone augmentation procedure [1]. Most of the morphological shifts in bone occur within the first 3 to 6 months after extraction [2], highlighting the importance of timely intervention to ensure the best possible outcome. Furthermore, an exacerbated resorption of bone, if unchecked, could lead to instability during routine oral functions, thereby impinging on the mastication, articulation, and the effective retention of dentures [3].

Notably, bone augmentation may be necessary when there is insufficient bone for implant placement, considering that the success of this procedure is heavily dependent on sufficient bone volume at the implant location [4].

### Membranes

In the context of bone augmentations, membranes (absorbable or non-absorbable) are used to facilitate Guided Bone Regeneration (GBR). A meta-analysis by Guo et al. [5] concluded that absorbable membranes presented a superior rate of successful regeneration when compared to non-absorbable membranes in GBR. Moreover, both the height and thickness of bone grafts were found to be greater with the use of absorbable membranes in comparison to non-absorbable counterparts, which may indicate them as confounding factors. Regarding safety, absorbable dental membranes demonstrated lower incidence of complications than non-absorbable ones.

An in vitro study [6] using Bovine Pericardium Membranes (collagen membrane) demonstrated that membrane thickness could possibly influence cell division and proliferation. The investigation employed optical density as a metric for comparing the quantity of metabolically active cells. Notably, a statistically significant difference was observed only after 24 h between membranes of 0.2 mm and 0.4 mm thickness. However, no statistically significant differences were observed at 72 h and at 7 days. No human studies were identified that support the correlation between membrane thickness and the quality or quantity of bone regeneration.

### Defect morphology

In a case-series of 28 patients [7], the initial bone morphology was identified as a factor influencing the outcome of GBR. Specifically, a bone concavity with a depth ≥1.03 mm and an angulation <155.30° were associated with a lower resorption rate of the grafted bone.

### Flap

A study focusing on Patient-Reported Outcome Measures (PROMs) [8] subsequent to GBR demonstrated that postoperative symptoms are most severe on the second day following the procedure. In addition, the oral health-related quality of life was significantly impacted by both the duration of surgery and the extent of flap advancement. Moreover, appropriate flap base width, characterised by a trapezoidal shape was recommended to reduce the risk of flap necrosis [9].

A systematic review [10] has highlighted that in plastic periodontal surgery, flaps with a thickness below 0.7 mm may have an adverse impact on flap vascularity.

### Tension-free flap

Effective bone augmentation procedures rely on adequate soft tissue coverage as it follows the original contour of the alveolar bone [11]. Insufficient soft tissue may lead to post-surgical complications, including soft tissue dehiscence and bone graft exposure [12, 13], implant failure and infection [14]. To ensure a successful bone coverage and regeneration, it is mandatory to achieve tension-free closure of the soft tissues [15]. This closure requires primary wound coverage during the healing phase to minimise the risk of infection and promote passive-tension-free flap closure [14, 16]. However, a significant amount of bone grafting material can make it difficult to achieve a tension-free primary closure, given the added bone graft typically has more volume than the available soft tissue the flap could cover, particularly in the case of bone block grafts [11]. These clinical settings require an extensive mobilisation and advancement of soft tissues, which may lead to increased swelling and trauma [16]. Clinicians employ the periosteal releasing incision (PRI) technique to achieve a tension-free flap, despite its potential to cause swelling and bleeding [16]. In addition, when attempting to restore soft tissue deficiency, clinicians may use various methods such as local flaps, pedicled flaps, free flaps, allograft, and alloplastic graft [11].

### Dehiscence (wound separation)

Wound dehiscence, graft exposure and infection are common complications of vertical ridge augmentation, with up to 38% for bone blocks, and 17% for GBR cases [17]. For bone regeneration, it is crucial to achieve a tension-free primary wound closure with low tension forces on the flap to establish an intact healing environment [13, 18]. However, it was estimated that wound dehiscence occurs in around 20% of complex augmentation cases [19]. One clinical study showed that flaps with tension greater than 0.25 N were prone to dehiscence, emphasising the importance of appropriate flap closure [20].

Previous studies in the field did not use tissue expanders and the rate of flap dehiscence was reportedly as high as 30% [21]. However, soft tissue expansion can increase the graft’s resistance against displacement, resulting in higher amounts of new tissue [22–24]. Therefore, it is important to have adequate soft tissue and tension-free wound closure for successful bone healing, which can be achieved through tissue expansion and proper flap closure techniques. Moreover, improving scaffold resilience against deformation and displacement can lead to a better outcome [25].

### Effect of soft tissue expansion on tissue flap and dehiscence

The use of tissue expanders can result in a tension-free flap and reduce the risk of wound dehiscence and subsequent exposures of bone grafts in complex augmentation procedures by increasing the available soft tissue [26, 27]. One study demonstrated that the simplicity of initial wound closure and healing are intimately connected [28]. Thus, managing flap tension when employing soft tissue expansion should be expected to contribute to primary wound healing. This approach can aid in the attainment of a contour change that conceals a scaffold with a closure free of tension [28].

The high incidence of wound dehiscence in control sites (8 out of 10) was a relevant outcome of bone augmentation procedure in one study [22]. Therefore, it is essential to find a way to minimise the occurrence of wound dehiscence during the healing phase to ensure the best outcome [22]

### Why use soft tissue expansion?

STE has been shown to have several benefits in oral surgery. It can create a tissue surplus that facilitates primary closure and increase the flap vascularity [24], reducing the demand for soft tissue grafts which eliminates donor site morbidity [29]. Moreover, STE can preserve the texture and colour of local soft tissue [29].

The surplus of soft tissue achieved during STE can be used to cover a bone graft [30] Additionally, STE not only causes tissue expansion but also enhances soft tissue vascularisation [31–33], which helps to mitigate the drawbacks of PRIs and reduces the impact on microcirculation following bone augmentation. Therefore, STE could be a valuable technique in oral surgery for achieving optimal soft tissue volume and preservation.

### How soft tissue expansion works?

The tissue expansion technique involves a Creep which results in the Biological Stretch [34]. The goal of this technique is to stretch tissue gradually, leading to growth and the formation of new cells without impacting the integrity of the original tissue [34, 35]. Expansion rate and expansion volume of the expander relies on the concentration and ion content of the surrounding tissue fluids as well as the components of the hydrogel inside the expander. This hydrogel is insoluble in water and absorbs the surrounding fluids causing the expander to swell [24, 27, 36].

Self-inflating tissue expanders are made up of an osmotic active hydrogel, a methylmethacrylate (MMA) core, a perforated N-vinylpyrrolidone membrane and a semi-permeable silicone shell. The expansion of the tissue is due to the hydrogel, which increases its volume through osmosis. The osmotic gradient ensures a continuous inflow of tissue fluid into the expander, increasing the volume of the expander simultaneously with the soft tissue growth by applying pressure that can reach approximately 235 mmHg (equivalent to 31.3kPa) [31, 37]. Noteworthy that rapid expansion will result in extreme increase in pressure which may cause tissue hypoxia or lead to perforation. Conversely, a gradually slow expansion will give the soft tissues more time to grow, stretch and adapt to the pressure exerted (Creep and Biological Stretch), reducing the likelihood of damage [38, 39].

### Rationale behind this review

The rational for conducting this review stems from a series of observations. Notably, a significant portion of complications in bone augmentation procedures relate to problems like dehiscence and graft exposure. The prevailing cause of these complications lies in the absence of adequate soft tissue to ensure tension-free closure of the surgical flap. An alternative approach involves employing STE, which generates an excess of soft tissue volume. The underlying concept is that this expansion technique could potentially reduce complication rates by providing additional soft tissue, thereby facilitating tension-free flap closure and reducing the risk of wound dehiscence and graft exposure. This review sought to examine the evidence from human studies to support this hypothesis. If proven true, the implementation of soft tissue expansion could eliminate the necessity for supplementary tissue graft augmentation surgeries. Furthermore, the surplus soft tissue resulting from this expansion technique holds promise for enhanced aesthetic outcomes, as it would harmonise with the natural colour and texture of the surrounding original soft tissue.

### Future of bone augmentation

Each grafting procedure has its own set of risks and benefits. No singular biomaterial or clinical technique can be deemed universally optimal [40], thus, clinicians must exercise caution when selecting an approach that yields favourable results while minimising complications.

Few papers [40, 41] have provided evidence in the literature suggesting that forthcoming advances may include the development of custom-made resorbable scaffolds and titanium meshes, in conjunction with tailored allogenic and xenogeneic grafts, to enhance bone regeneration outcomes.

The trajectory of innovation in bone augmentation has many potential avenues, making it challenging to extrapolate the future based on a limited number of studies. Nevertheless, it is clear that the overarching objective of these innovations in the realm of surgical dentistry is the accomplishment of an optimal approach for repairing soft tissue deficiencies, implementation of ideal flap designs, alongside the judicious selection of appropriate membranes and graft materials.

### Objectives

The present review aims to assess the current literature regarding the effectiveness of soft tissue expansion (STE) when using self-inflating osmotic hydrogel tissue expanders (SOHTEs) in gaining surplus soft tissue for primary wound coverage prior to bone augmentation, as well as in mitigating the incidence of complications

## Methods

The PRISMA checklist was followed for reporting this review. The PICO framework was used to structure the reporting of eligibility criteria:

(P) Population - Adult patients with compromised alveolar bone and hence requiring bone augmentation; (I) Intervention - STE using SOHTEs; (C) Comparisons: no STE or no comparison; (O) Outcomes - Successful primary wound coverage and complication rates (Primary outcomes), soft tissue volume and bone volume (Secondary outcomes).

### Search strategy

PubMed and Google Scholar were employed as primary platforms for conducting the search for articles included in this review. The following table delineates the key search terms used for articles retrieval. The databases were last searched in March 2023 (Table 1).

**Table 1:** Summary of search terms in each database.

PubMed Search	Google Scholar Search
(soft*[Title] OR osmo*[Title] OR self*[Title] OR hdro*[Title] OR periosteal*[Title] OR submuco*[Title]) AND (tissue*[Title]) AND (expan*[Title])	allintitle: (soft OR hydrogel OR osmotic OR self-inflating OR self OR self-filling) (tissue OR tissues OR inflating OR filling) (expansion OR expander OR expanding OR expand)

Last searched on: March 2023

### Study selection

We included human studies specifically reporting SOHTEs for STE within the oral cavity and focused on studies involving adult subjects. We excluded studies that did not satisfy these specific conditions or matched the exclusion criteria. The eligibility criteria ensured that the selected studies were directly relevant to the use of SOHTEs for STE in adult human populations within the oral cavity (Table 2).

**Table 2:** Summary of inclusion and exclusion criteria.

Inclusion criteria	Exclusion criteria
• Randomized Controlled trials (II)^a^ • Cohort studies (III)^a^ • Case-control studies (IV)^a^ • Case-series (IV)^a^ • Not restrictions on date • No restrictions on sample size • No restrictions on gender • No restrictions on bone augmentation technique or materials	• Case reports (V)^a^ • Animal studies (V)^a^ • Laboratory studies (V)^a^ • Reviews, editorials, letters, commentaries, or conference abstracts • Systematic reviews and meta-analyses • Non-English articles • Studies not using SOHTEs for STE • Studies using SHOTEs extraorally • Duplicate Studies

^a^Studies were categorised using the Oxford Centre for Evidence-Based Medicine Levels of Evidence classification system [34].

Study selection was conducted by two independent reviewers (AGE, RS) in the following stages: (1) Initial screening of potentially suitable titles and abstracts against the inclusion criteria to identify potentially relevant papers. (2) Screening of the full papers identified as possibly relevant in the initial screening. (3) Studies were excluded if not meeting the inclusion criteria. Following the screening of titles and abstracts, the studies included by both reviewers were compared. In case of a disagreement between reviewers, the decision about study eligibility was made by trying to reach a consensus between the two reviewers. For continued disagreement, a third reviewer (HH) judged study inclusion.

### Data collection

In relation to each investigated study, data collection was undertaken independently by two reviewers (AGE, RS). Another author (INP) reviewed extracted data and resolved any discrepancies.

(1) **Study publication details**: Authorship, year of publication, and country of origin. (2) **Study characteristics**: Demographic variables encompassing sex distribution, age demographics, and duration of follow-up. (3) **Study methodology**: SOHTE brand, if a silicone shell was component in the SOHTE used, anatomical site of intervention, location of SOHTE implantation, dimensions of SOHTE, and duration of the expansion process. The selected studies were categorised using the Oxford Centre for Evidence-Based Medicine Levels of Evidence classification system [42]. (4) **Study outcomes**: Assessments of soft tissue attributes, quantitative evaluations of bone augmentation and number of complications encountered. We planned to calculate the risk ratio of complications to determine improvements when using STE. However, if there are significant lack of controls, we planned to calculate an overall complication rate of all studies and compare it to the overall complication rate of non-STE bone augmentation studies.

Our research methodology encompassed the intention to compute the risk ratio pertaining to complications, aiming to discern enhancements associated with the use of STE. Nevertheless, in instances where a significant lack of control groups was evident, an alternate approach was devised. This involved calculating an aggregate complication rate derived from all encompassed studies, facilitating a comparative analysis against the overall complication rate documented within non-STE bone augmentation studies.

### Data preparation

In instances where a specific data point was entirely absent, we systematically documented and presented this absence as “Not Reported” (NR) in our analysis. When the complication rate value was unreported within a given article, we undertook a calculation employing the total participant count and the observed frequency of complications to derive this metric. Regarding continuous variables, such as age, when distinct values were provided for each respective group, we aggregated and subsequently presented the combined mean and standard deviation values as part of our analysis.

### Risk of bias

The Risk of Bias 2 (ROB2) assessment tool was used to assess risk of bias for RCTs (Level II). For each study the overall bias was given based on the highest bias score for each decision category. For example, if the highest score of ‘Moderate’ was estimated for one or more decision categories, then the overall bias was considered ‘Moderate’.

### Data analysis, statistical methods, and data visualisation

We synthesized evidence narratively as well as graphically. Plots have been shown to be an effective way to summarise evidence. RS produced the plots after the data collection and analysis was complete using the plotly library in python programming language. The plots produced are sunburst, bar charts, and scatter plots. Risk of Bias 2 (RoB2) assessment tool [43] was used by RS to create the risk of bias summary tables. We anticipated that a meta-analysis could not be undertaken due to the heterogeneity of interventions, settings, study designs, outcome measures and lack of controls.

## Results

### Studies included

**Fig. 1:**
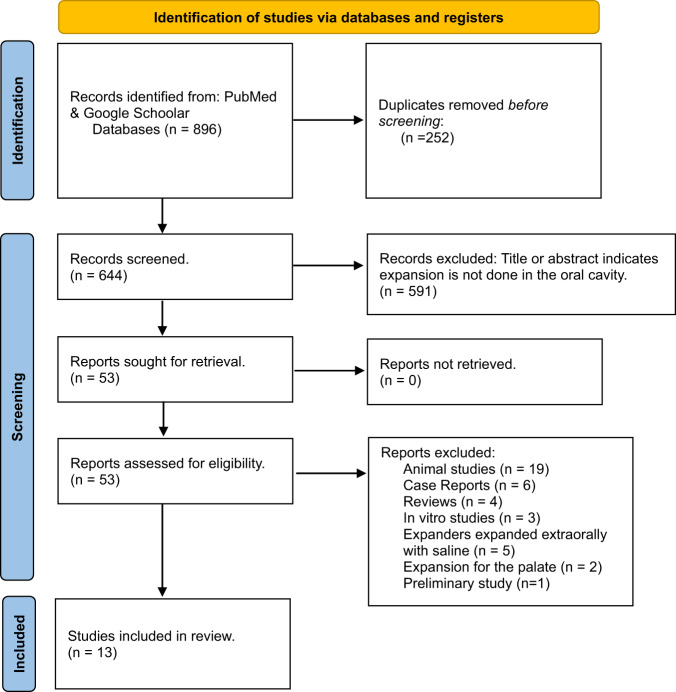
PRISMA flowchart. A flowchart demonstrating the identification, screening and the inclusion process of the SOHTE articles in this review.

**Fig. 2:**
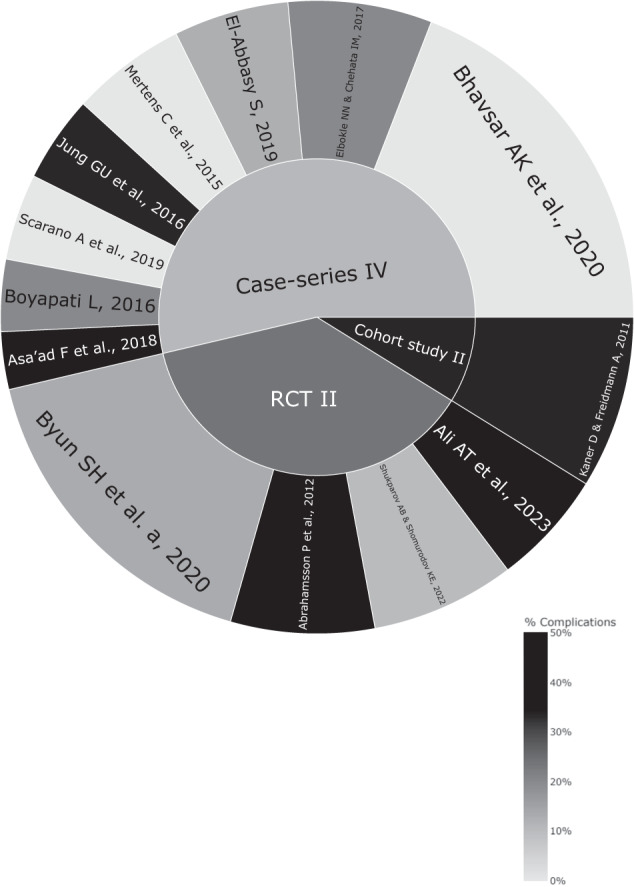
Complication rates of SOHTE studies. A sunburst plot demonstrating the complication rate for each included study and their identified study type.

Figure 1 shows the PRISMA flowchart [44] representing study selection and inclusion. The initial search resulted in 896 papers for all databases combined. This was trimmed down to 644 after duplicates were removed. Following the first-stage screening of titles and abstracts, 53 articles (considered potentially suitable by at least one reviewer) qualified for full-text screening. After full-text reading, 14 articles met the defined inclusion criteria, and 39 papers were excluded (see Fig. 1 for reasons for exclusion). However, two articles [32, 33] shared participants in their samples. Considering article quality and relevance to our paper, the preliminary study [33] was the selected study to be included for analysis in our evidence-based review to avoid the potential bias arising from the overlapping cohort of patients. Figure 2 shows the articles in accordance with their study type (case-series vs RCT vs cohort) along with their complication rates.

### Study characteristics

**Fig. 3:**
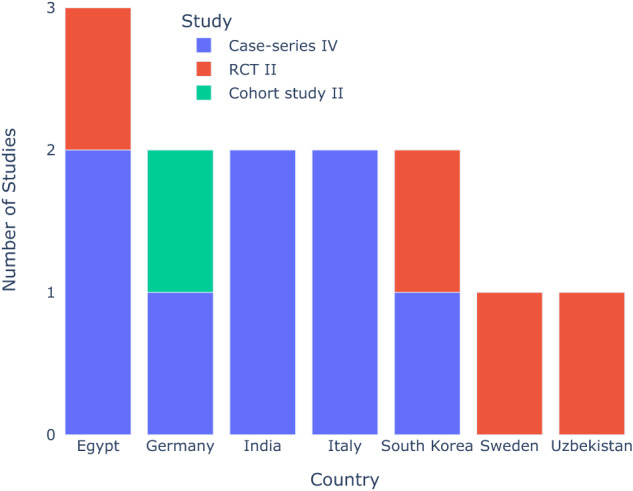
Distribution of SOHTE studies across countries. A bar chart demonstrating the number of studies with their corresponding level of evidence in each country.

Table 3 reports the studies and their characteristics, which included 4 RCTs, 1 cohort studies, 8 case-series. The table shows the authors, year of publication, country of publication, study type, level of evidence, and interventions. Studies were classified as level of evidence II and IV, with RCTs being at level II and a cohort study also at level II due to its quality and large effect in results. The case-series were classified at level IV. Follow-up interval ranged between 4-12 months. There were slight variations in the number of participants between the different case-series studies, with RCTs expressing on average a higher number of participants. Nevertheless, the total number of patients undergoing STE in experimental groups in RCTs was comparable to the total in the case-series. Although the RCTs were placed at level II, few of them have limited strength, mainly attributed to their limited sample sizes within the STE groups, encompassing 10 or fewer sites. On the other hand, case-series were categorised at level IV. The study conducted by Kaner & Friedmann [22], which incorporated a split-mouth design, demonstrated robust methodological quality, positioning it as a high-calibre cohort study equivalent to a RCT classified at level II (Fig. 3).

**Table 3:** Summary of studies characteristics.

Author, Country, Year	Study type, level of evidence, follow-up (months)	Population characteristics	Intervention
Kaner & Freidmann, 2011 [22], Germany	Cohort study, II, 4–6 months	Total pt: 12 Total sites: 24 Total STE sites: 12 males: 3 females: 9 Age: 45 (21–73)	STE + VBA with autogenous bone (*n* = 12) vs VBA with autogenous bone (*n* = 12)
Abrahamsson et al. 2012 [23], Sweden	RCT, II, 6 months	Total pt: 20 Total sites: 20 Total STE sites: 10 males: 14 females: 6 Age: 26 (18–55)	STE + bone graft from mandibular ramus + Ti mesh + CM (*n* = 10) vs mandibular ramus + PRI (*n* = 10)
Mertens et al., 2015 [45], Germany	Case-series, IV, 4 months	Total pt: 8 Total sites: 11 Total STE sites: 11 males: 3 females: 7 Age: 49 (26–74)	STE + VBA/HBA with autogenous bone
Jung et al., 2016 [46], South Korea	Case-series, IV, 36 months	Total pt: 6 Total sites: 6 Total STE sites: 6 males: 3 females: 3 Age: 54 (47–63)	STE + autogenous graft + xenograft + CM
Elbokle & Chehata, 2017 [50], Egypt	Case-series, IV, 4 months	Total pt: 10 Total sites: 10 Total STE sites: 10 males: 6 females: 4 Age: 32 (18–60)	STE + VBA with xenograft
Asa’ad et al., 2018 [48], Italy	Case-series, IV, 9 months	Total pt: 4 Total sites: 5 Total STE sites: 5 males: 1 females: 3 Age: 53.75 (44–60)	STE + bone harvested from site + DBB + d-PTFE/ CM
Scarano et al., 2019 [51], Italy	Case-series, IV, 12 months	Total pt: 6 Total sites: 6 Total STE sites: 6 males: 4 females: 2 Age: (18–35)	STE + GBR + sinus augmentation
Byun et al., 2020 [32], South Korea	RCT, II, 12 months	Total pt: 46 Total sites: 46 Total STE sites: 23 males: 24 females: 22 Age: 57.63	STE + d-PTFE Ti reinforced + + xenograft + PRI (*n* = 23) vs d-PTFE Ti reinforced + + xenograft + PRI (*n* = 23)
Ali et al., 2023 [52], Egypt	RCT, II, 6 months	Total pt: 15 Total sites: 15 Total STE sites: 8 males: 1 females: 15 Age: (22–54)	STE + HBA (*n* = 8) vs PRI + HBA (*n* = 7)
El-Abbasy, 2019 [49], Egypt	Case-series, IV, 2 months	Total pt: 8 Total sites: 8 Total STE sites: 8 males: 6 females: 2 Age: 51.7 (44–61)	STE + allogenic bone
Boyapati, 2016 [47], India	Case-series, IV, 6 months	Total pt: 5 Total sites: 5 Total STE sites: 5 males: 5 females: 0 Age: 36.2 (26-53)	STE + autogenous block + DMBM
Bhavsar et al., 2020 [53], India	Case-series, IV, 6 months	Total pt: 10 Total sites: 26 Total STE sites: 26 males: 5 females: 5 Age: 35.2 (25–40)	STE
Shukparov & Shomurodov, 2022 [37], Kazakhstan	RCT, II, 6 months	Total pt: 10 Total sites: 10 Total STE sites: 10 males: 25 females: 35 Age: 40 (20–75)	STE + VBA

*STE* Soft Tissue Expansion, *VBA* Vertical Bone Augmentation, *HBA* Horizontal Bone Augmentation, *Ti* Titanium, *CM* Collagen Membrane, *PRI* Periosteal Releasing Incision, *d-PTFE* dense polytetrafluoroethylene, *DBB* Demineralised Bovine Bone, *GBR* Guided Bone Regeneration.

Only the study by Abrahamson included smokers, which demonstrated an effect on the results, both on soft tissue gain and magnitude of bone gain after 6 months. Most studies included both female and male participants. However, some studies reported an unequal sample according to the gender; either there was considerably more males or females. In terms of age ranges of samples, the largest number of papers refer to 20–65 years old, while overall the participants fell between the ages of 18–74.

The majority of the studies reported the defect sites in both the maxilla and the mandible [22, 32, 33, 37, 45–48]. The remaining assessments only included defect sites in the posterior area of the mandible [49, 50], followed by the maxillary [23, 51, 52]and mandibular anterior location [53], respectively. The inclusion criteria adopted by many studies encompassed patients presenting with deficiencies in their soft tissue. Thus, referring to clinical scenarios, wherein bone augmentation procedures carry an increased susceptibility to complications and a less favourable prognosis compared to typical cases.

### Countries and levels of evidence

Studies were conducted in Europe (Germany, Italy and Sweden), Asia (South Korea, India, Kazakhstan) and Africa (Egypt). The leading country with 3 studies is Egypt, with the bottom being Sweden and Kazakhstan with 1 study each, the rest being at two studies each. Among these regions, Egypt and South Korea emerged as the primary contributors, each conducting one RCT and case-series studies with high quality studies. Conversely, Sweden and Kazakhstan demonstrated lower participation, with just one study each. The remaining nations yielded two studies each (Fig. 3).

### Risk of bias analysis

The Risk of Bias 2 (RoB2) assessment tool [43] was employed to appraise the degree of bias in each RCT. For Domain 1 (D1) Shukparov AB and Shomurodov KE have not provided sufficient detailed information about the participants’ baseline characteristics. This lack of information makes it challenging to confirm if there were no significant differences among participants that could indicate a fair randomization process. For Domain 2 (D2), which investigates whether any deviations from the planned interventions could potentially cause bias, no instances were identified in any of the studies analysed. Regarding Domain 3 (D3). Shukparov AB and Shomurodov KE have not clearly stated if all participants completed the entire trial, raising uncertainty about the completeness of the data shared. This lack of clarity makes it difficult to determine whether all relevant information from all participants has been accounted for. The absence of outcome assessor blinding is a factor concerning Domain 4 (D4) and all the analysed studies. This absence of blinding introduces some reservations regarding the reliability and potential bias in the outcome measurements conducted in these studies. As for Domain 5 (D5), Shukparov AB and Shomurodov KE’s studies, despite conducting measurements of radiographic results, these findings have not been reported. This omission raises the possibility that outcomes were selected for reporting based on certain criteria, which could introduce a bias in the presentation of results.

**Fig. 4:**
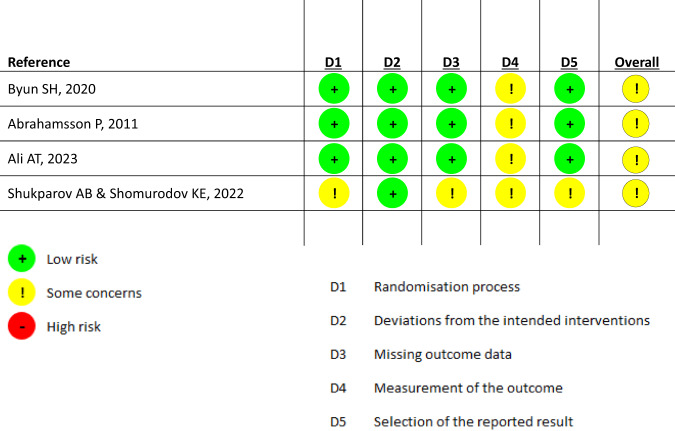
ROB2 results for SOHTE studies. Decision matrix demonstrating the risk of bias identified in each decision category for RCTs using SOHTEs.

**Fig. 5:**
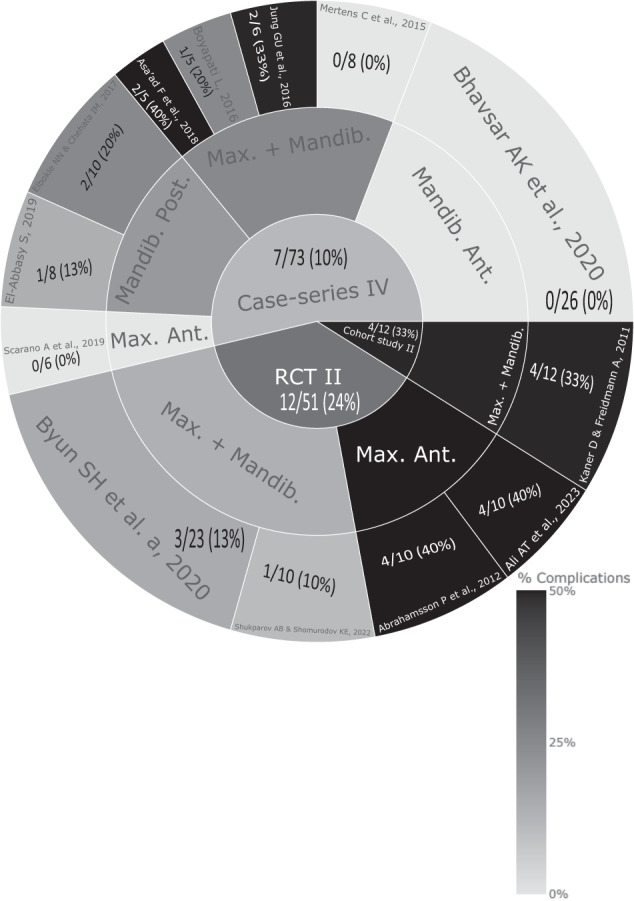
Sunburst plot presenting the complication rate for each study type and its corresponding studies with the soft tissue expansion sites Max. Maxilla, Mandib. Mandible, Post. Posterior, Ant. Anterior.

Across all RCTs under consideration, some of the specific domains addressed by the ROB2 raised concerns, including the absence of blinding among assessors regarding the reporting of radiographic outcomes. Moreover, a high bias was recognised in the assessment of “Soft Tissue Volume Gain”, given the absence of a placebo group and the non-blinded status of assessors. It is noteworthy, however, that the level of bias pertaining to “Complication Rates” remains nominal, since the involvement of assessors is unlikely to exert an influence upon the rates of complications observed between patient groups (Figs. 4 and 5).

### Complications rate

The assessment of complications within the studies was conducted through either direct observation or structured evaluations employing predefined checklists, aimed at identifying undesired outcomes such as wound dehiscence, perforation, and graft exposure.

A predominant proportion of the retrieved studies adopted a case-series design, which lacked control groups. Consequently, the computation of risk ratios was precluded due to this absence of comparative data. As a viable alternative, we opted to consolidate and synthesise an aggregate overall complication rate based on the available information.

**Fig. 6:**
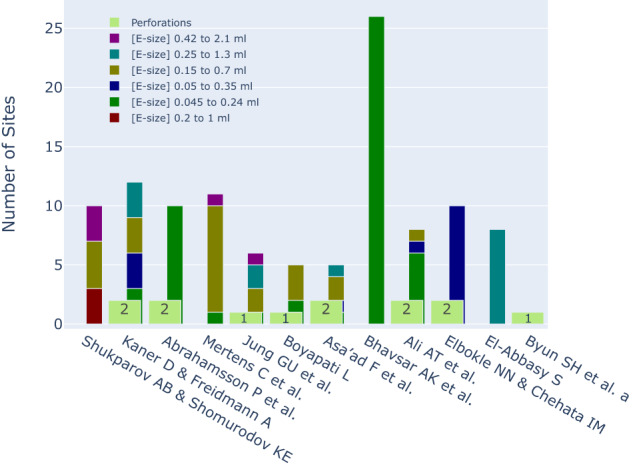
Expander perforation occurrences and sizes of expanders in SOHTE studies. A Bar chart demonstrating the occurrence of expander perforation and the size of expanders used in each included study. [E-size] X to Y = expander size with initial volume of X and final Volume of Y.

The observed complication rates displayed variability, with a substantial proportion of these complications being attributed to perforations (Table 4 and Fig. 6).

**Table. 4:** A summary of complication rates of 2011–2023 SOHTE studies.

Complication	Complication incidence (incidence out of 140 sites)
Perforation	9.3% (13)
Graft exposure	2.1% (3)
Infection/ fistula	1.4% (2)
Dehiscence	1.4% (2)
Paraethesia	1.4% (2)
Displacement	0.7% (1)
Expander rupture	0.7% (1)
Overall	17.13% (24)

#### Antibiotic and antiseptic protocols

**Fig. 7:**
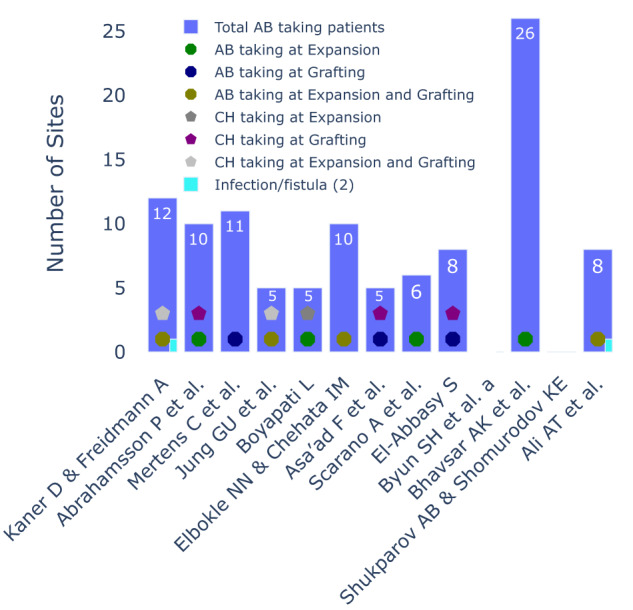
Infection occurrences with antibiotic and antiseptic protocols in SOHTE studies. A Bar chart demonstrating the occurrence of infection occurrences with antibiotic and antiseptic protocols in each included study. AB antibiotics, CH chlorhexidine.

Across various studies concerning SOHTEs conducted between 2011 and 2023 there was great disparity in the prescribed antibiotic (AB) regimens and during the phases at which they were prescribed (expansion, *n* = 4 [23, 47, 51, 53]; grafting, *n* = 3 [24, 45, 49]; or both, *n* = 4 [22, 46, 50, 52]). Three studies did not use antibiotics at all [32, 33, 37]. Antiseptic employment predominantly centred on chlorhexidine (CH), being administered at different stages (expansion, *n* = 1 [47]; grafting, *n* = 4 [23, 24, 33, 49]; or both, *n* = 2 [22, 46]). Seven studies did not use chlorhexidine [32, 37, 45, 50–53]. It merits mention that a mere two studies reported instances of infection, contributing to a cumulative total of two infections among the aggregated 140 sites across all investigations (Fig. 7).

#### Type of expander

The studies included two distinct types of SOHTE brands, with the predominant choice being the Osmed expanders and only three studies reporting the use of Osstem expanders. While for Osmed it was determined the timeframes for the expansion phase relating to the corresponding expander size, Osstem expanders lack such specific temporal guidelines.

#### STE implantation location

According to the included studies, STE was placed either subperiosteally or submucosally. The vast majority reported placing it, subperiosteally, with only 3 studies adopting the submucosal approach.

#### Defect site

The conducted investigations have reported the application of STE on both mandibular and maxillary regions [22, 32, 33, 37, 45–48]. Some studies focused on the upper arch [23, 51, 52]. In contrast, other studies concentrated on the lower arch [49, 50, 53].

#### Expansion duration

The duration of expansion ranged between 10 and 60 days, with a prevailing trend toward a 4-week expansion duration which was the most frequently employed.

#### Expander size

Across the included studies, a range of expander sizes were employed and yielded final volumes of 0.045, 0.05, 0.15, 0.2, 0.25, and 0.42 ml. While smaller expander sizes were more frequently utilised compared to larger sizes, it was acknowledged that the majority of studies omitted the explicit reporting of the specific expander size associated with each respective perforation (Fig. 6).

### Radiographic results

**Fig. 8:**
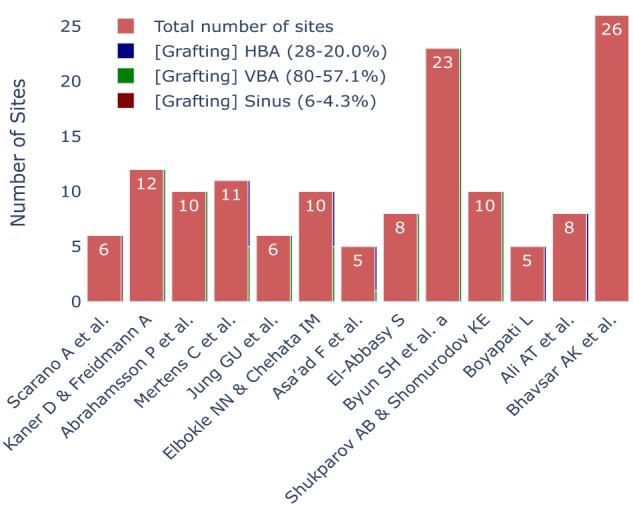
Total number of sites with the number of sites employing different augmentation procedures. A Bar chart demonstrating the total number of sites with the distribution of sites employing vertical bone augmentation, horizontal bone augmentation and sinus lift procedures. VBA vertical bone augmentation, HBA horizontal bone augmentation.

The reporting of bone gain was limited to a subset of studies. As depicted in Fig. 8, the outcomes revealed that most participants underwent vertical bone augmentation procedures (57.1%, 80 out of 140 sites), followed by horizontal augmentation procedures (28%, 28 out of 140 sites), and sinus lift procedures (4.3%, 6 out of 140 cases), respectively.

### Soft tissue volumes

**Fig. 9:**
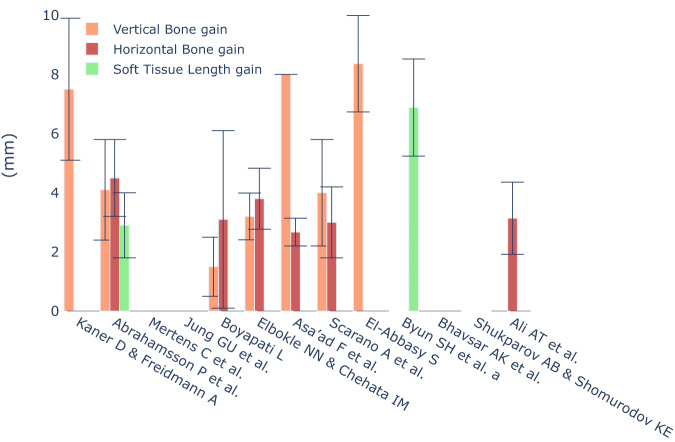
Radiographic results and soft tissue length gain reported in SOHTE studies. A bar chart demonstrating the radiographic bone gain (mm) and soft tissue length gain (mm) reported in each study. An empty bar means the study did not report these variables.

**Fig. 10:**
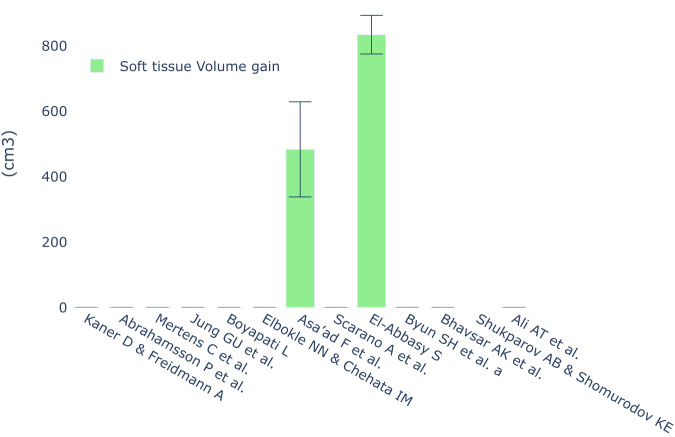
Soft tissue volume gain reported in SOHTE studies. A bar chart demonstrating the soft tissue volume gain (cm3) reported in each study. An empty bar means the study did not report soft tissue volume gain.

Regarding the quantification of soft tissue volumes, most studies employed methods involving casts, optical scanning technologies, and computer-aided software to track alterations in soft tissue [24, 32, 33, 49]. Only one study implemented advanced 3D measurement equipment to gauge soft tissue augmentation, reporting the most prominent dimensional increase achieved in the context of soft tissue expansion [23] (Figs. 9 and 10).

## Discussion

From this review based on 13 studies and 136 participants with 140 STE sites, it seems the data exploring the use of SOHTE on humans goes back to 2011. However, the evidence has not yet been able to establish definite guidelines for research and clinical practice tailored to each individual case, which indeed gives rise to several inquiries regarding the optimal dimensions suitable for distinct anatomical sites, the risk-benefit of subperiosteal versus submucosal implantation for SOHTEs, the ideal duration of expansion corresponding to specific expander sizes and the best method and location of implantation. Furthermore, considerations extend to the recommended protocols for antibiotic and antiseptic administration, the potential influence of variables such as age and gender on the efficacy of soft tissue expansion, and the most effective approach to bone augmentation when employed conjointly with the practice of soft tissue expansion.

In light of the current state of knowledge, the results presented in this review demonstrated that the available information regarding the use SOHTEs for STE remains limited and primarily anecdotal. Only two distinct types of SOHTE brands were identified among the included evidence - Osmed and Osstem expanders. The former was the most popular brand, which offered recommendations for expansion duration. However, Osmed´s guidelines were based on in vitro experiments and the absence of corresponding human data hampered our ability to confidently determine the optimal expansion duration for clinical applications. As for the Osstem expanders, we found that the manufacturer’s endorsement of surgical templates to determine expander size hilights the lack of standardised methodologies in this field. Additionally, the closure of the Osmed brand raises questions about the availability and continued development of SOHTEs for future research and clinical use. It seems therefore that Osstem brand may become the pivotal player in this context, and potentially the sole remaining manufacturer shaping the course of advancement in the field of STE.

### Results against current knowledge

Although we need to exercise caution in interpreting the findings of this review, they appear to indicate that studies focused on STE employing SOHTEs have demonstrated notably reduced occurrences of dehiscence (1.4%, 2/140 sites) and paraesthesia (1.4%, 2/140 sites). However, there are a couple of important questions worth further exploration. First, according to the results of this review, combined perforations rates (9.3%, 13/140 sites) accounted for more than half of the complications (54%, 13/24 complications). Our understanding is that perforation-related incidents are deemed avoidable, therefore, an effective mitigation of perforation complications could have consequentially yielded a marked reduction in overall complication rates.

Additionally, we found challenging to evaluate infection-related data extracted from the 2011 to 2023 studies concerning SOHTE, particularly when employing diverse antibiotic and antiseptic protocols, which prevented to unequivocally establish a causal relationship or identify an optimal protocol for mitigating infection rates. Hence, it is evident that studies with representative sample sizes and stringent methodological controls are necessary to reveal the dynamics underlying infection incidence in relation to the administration of antibiotics and antiseptics within the context of STE procedures.

Regarding the expansion configuration, Osstem exhibited characteristics related to consistent kinetics [32]. However, the available evidence does not provide robust grounds to endorse the superiority of any brand, particularly given the relatively recent introduction of Osstem expanders, which has been featured solely in two independent studies [32, 33, 37]. Consequently, definitive conclusions regarding the comparative safety and efficacy of these two SOHTE types remains unclear.

We also do not have enough evidence to suggest which implantation location is best at reducing complication rates. Weak evidence, based on studies with small sample sizes and some concerns with bias, seems to suggest that the submucosal technique may be advantageous for total bone gain after augmentation, [22] while the subperiosteal procedure may be favourable to reduce the complication rate. However, considering the absence of strong correlations and the limited size of the comparative submucosal subgroup, it is difficult to confidently establish a connection between implantation location and the subsequent complication rates. Hence, it is unclear whether STE implantation location may be important for the clinical decision-making process. Moreover, we do not have accessible individual patient data to explore potential correlations between complications and the specific defect sites within oral cavity.

Our analysis indicated that the manufacturer’s guidelines proposed the ideal duration for expansion (most commonly 4 weeks). However, the available clinical trials provided limited evidence substantiating the most favourable expansion duration. This was contingent upon distinct expander configurations (shape and size) to achieve optimal tissue volume augmentation while at the same time mitigating the risks of perforation and other associated complications. Future RCTs should include data on the expander size, configuration, and expansion duration to each complication episode. This approach is crucial to facilitate prospective meta-analyses to interpret outcomes and determine the optimal duration of expansion. Moreover, considering the insufficient data, a sizable RCT emerges as a requisite for the exploration of this subject matter.

Given the limited number of trials providing information regarding the expander size when reporting expander perforations, it was also not possible to establish a definitive correlation between expander size and the incidence of perforations. We found similar methodological challenges when assessing the relationships between the rate of expander perforations and several other factors, including expansion site, expansion duration, gender, and age. For that reason, it is strongly recommended that forthcoming research meticulously report individual patient data across all variables, which will facilitate the amalgamation of comprehensive datasets and empower thorough analyses to reveal potential interrelationships among variables. The future trajectory of studies in this domain should therefore adhere to a rigorous approach, which will allow a comprehensive assessment of any discernible connections that may exist between expander dimensions and the incidence of perforations. Nevertheless, although the existing data may not currently support the establishment of a statistically significant association between expander variables and the incidence of perforations, the literature consistently recommends avoiding overly large expanders, utilising prolonged expansion durations, or siting expanders near the incision line [22, 23, 32, 33, 52]. These recommendations are anecdotally formulated with the intent to reduce the perforation rate.

Another important finding from this review was that the radiographic outcomes of bone gain in studies involving soft tissue expansion were comparable to those observed in standard bone augmentation studies. While few of the soft tissue studies indicated a greater increase in bone gain compared to the standard studies, it is important to note that these findings were based on studies with limited sample sizes and potential biases. Therefore, the evidence is not yet sufficient to conclusively assert that soft tissue expansion prior to bone augmentation leads to significantly higher bone gain (Figs. 9 and 10).

**Fig. 11:**
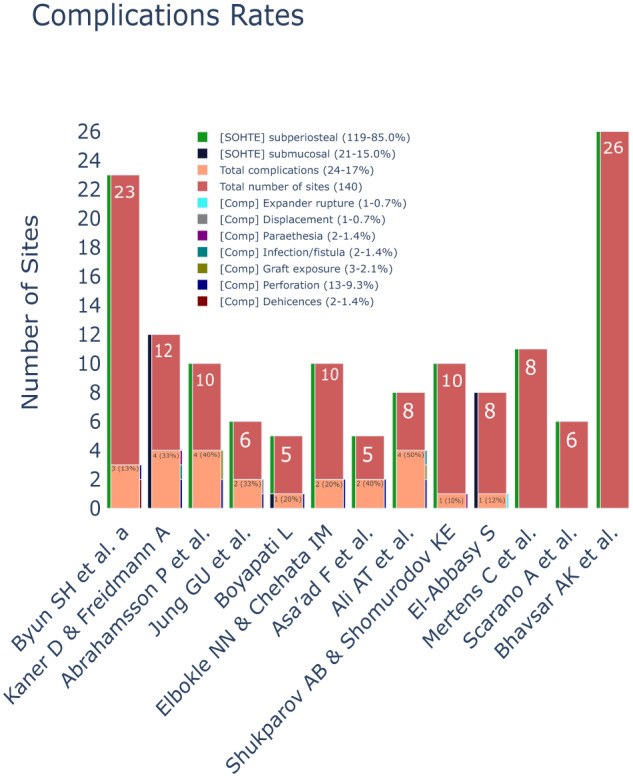
Overall complications incidences and the occurrences of the specific types of complications in SOHTE studies. A bar chart demonstrating the total sites, overall complication incidence, the occurrence of specific types of complications and the subperiosteal vs the submucosal implantation of expanders in each study. Comp complication.

Despite these challenging circumstances, SOHTEs demonstrated a low dehiscence and parethesia  rate of 1.4% each in comparison to investigations involving conventional STE techniques (Fig. 11), which indicated dehiscence incidences of 6.7% (15/221 sites) [54] and 12.5% (10/80 sites) dehiscence along with 10% (8/80 sites) temporary neurosensory disturbances [55]. Notably, a substantial proportion of subjects within the STE cohorts exhibited compromised soft tissue volume, thereby rendering the attainment of primary wound closure with tension-free flaps a more challenging goal, and consequently increasing the risk of dehiscence.

**Fig. 12:**
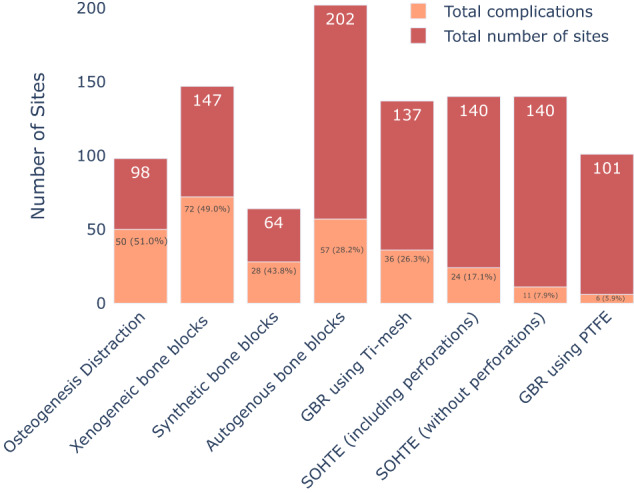
Combined complication rate of SOHTE studies vs combined complication rates of non-SOHTE studies. A bar chart demonstrating the total sites and total complications of SOHTE studies (with and without perforation incidences) and studies which did not use SOHTEs or employ STE prior to augmentation.

This provides important evidence of the benefits of STE in cases characterised by deficient or suboptimal soft tissues, circumstances that could otherwise escalate the vulnerability to wound dehiscence. We found evidence that this strategic approach results in an excess of soft tissue, facilitating the achievement of primary coverage. One systematic review [17], incorporating diverse bone augmentation techniques in its analysis, has merged the rates of complications associated with approaches devoid of STE (as depicted in Fig. 12). The results illustrate that the employment of STE is linked to a 17.1% complication rate, which is considerably lower than the complication rates observed across various bone augmentation studies conducted without utilising STE. It is noteworthy that this reduction is consistent across most techniques, with the exception of GBR with the use of polytetrafluoroethylene (PTFE), which reported a comparably lower complication rate of 5.9% (6 out of 101 sites) (Fig. 12).

Additionally, findings from STE studies suggest that it may therefore be appropriate to adopt meticulous measures to reduce perforation-related complications. These measures encompass the utilisation of surgical templates to select an appropriate expander size, employing expanders characterised by a consistent expansion rate, optimising expansion duration, situating the expander at a distance from the incision line, and advising patients to reduce the use of removable partial dentures (RPDs) during the expansion phase. If the risks associated with perforations can be successfully mitigated, the ensuing complication rate may decrease to 7.9% (11 out of 140 sites), aligning closely with the complication rates observed in GBR procedures with PTFE in the devoid of STE (Fig. 12).

More comprehensive data may help to draw definitive conclusions or establish standardised protocols for the application of SOHTEs in STE procedures. This highlights the necessity for robust, well-designed clinical studies that can provide substantiated guidelines for the use of SOHTEs in STE, considering variables such as expansion durations, expander sizes, and associated complications. Until such evidence is generated, clinical practitioners are left to navigate the use of SOHTEs based on the available anecdotal recommendations, necessitating cautious and informed decision-making in their applications.

### Implications for clinical practice and research for future directions

In outlining potential avenues for future investigations, it is important to acknowledge that the current body of evidence does not unequivocally endorse the routine use of soft tissue expansion (STE) prior to bone augmentation in typical clinical cases. However, moderately robust evidence does indicate the potential utility of STE in scenarios characterised by deficient soft tissue. To advance the field, forthcoming research with large sample size should aim to investigate any additional benefits that might arise from the implementation of STE in common cases, and also delve into the potential correlations between various variables and complication rates. Irrespective of the specific hypothesis being tested, it is imperative that study designs incorporate a comprehensive set of measurements and observations, encompassing parameters such as soft tissue gain, radiographic bone gain, patient demographics, defect site, duration and configuration of expansion, expander implantation methodology, and the incidence of complications for each individual patient. This thorough approach will facilitate robust quantitative analyses of outcomes, enabling the potential integration of results with those of other studies in subsequent investigations.

### Limitations of this review

As outlined in the search strategy, we limited publications to English language and hence it is possible that some evidence may have been missed.

In one sense, the eligibility criteria within the current study were inclusive (e.g., we included studies with or without control groups). However, this review was not restrictive in terms of the quality of evidence and could not, therefore, provide a robust evaluation of effectiveness.

Because of time constraints, single review author rated risk of bias and we introduced some risk of error. Moreover, the protocol for the present review was not registered online which does not allow a verification that review methods were carried out as planned. Nevertheless, we are confident that none of these methodological limitations have impacted the overall conclusions of this review.

While a total of 13 studies exploring the use of SOHTEs for STE have been conducted within (a span of 12 years), most of these investigations fall within Level IV, characterized by case-series designs without controls and inheriting limitations in terms of quality and substantial bias risks. Furthermore, all of the identified RCTs were classified at Level II exhibited some bias risks. Despite these weaknesses, we have chosen to incorporate and present their findings due to the valuable insights they offer into the subject matter. This inclusion serves as means to inform and guide forthcoming research. It is important to recognise that our efforts were directed towards using the existing evidence, even while acknowledging its inherent limitations. This recognition is fundamental as it highlights the necessity of engaging with the available information to drive well-informed strategies for future research. It is noteworthy that these preliminary findings hint at the promise associated with the use of SOHTEs and STE. Thus, more robust RCTs are required to expand upon these initial indications.

## Conclusion

STE investigations have demonstrated the advantageous role of SOHTEs in reducing complication rates associated with bone augmentation procedures when dealing with insufficient soft tissue. Nonetheless, the evidentiary basis for the STE supplementary benefits in scenarios marked by adequate soft tissue volume remains unclear.

Attentive procedural precautions can play a pivotal role in minimising the risk of expander perforations, thereby affording an avenue to further diminish the overall complication rate.

Although the collective quality of evidence assessing the healing and complications of STE using SOHTEs prior to bone augmentation is not optimal, the findings emphasised the need for additional RCTs of heightened quality to corroborate and solidify the conclusions and offer an important direction for future research. Prospective studies should diligently document individual patient and expander-specific data encompassing complications, soft tissue augmentation, and bone augmentation. This comprehensive dataset will subsequently facilitate future meta-analyses, thus facilitating a more precise exploration of the relationships among these variables. The gain of such robust data will eventually underpin the formulation of well-defined guidelines pertaining to the judicious use of SOHTEs for STE.

## Data Availability

The data that support the findings of this study are available from the corresponding author upon reasonable request.
